# 3-(2-Amino-1-methyl-4-oxo-4,5-dihydro-1*H*-imidazol-5-yl)-5-fluoro-3-hydr­oxy-1-methyl­indolin-2-one methanol hemisolvate

**DOI:** 10.1107/S1600536809043797

**Published:** 2009-10-31

**Authors:** Narsimha Reddy Penthala, Thirupathi Reddy Yerram Reddy, Sean Parkin, Peter A. Crooks

**Affiliations:** a Department of Pharmaceutical Sciences, College of Pharmacy, University of Kentucky, Lexington, KY 40536, USA; b Department of Chemistry, University of Kentucky, Lexington, KY 40506, USA

## Abstract

In the title compound, C_13_H_13_FN_4_O_3_·0.5CH_3_OH, mol­ecules are packed in the crystal structure by a series of O—H⋯N, N—H⋯O, N—H⋯F and O—H⋯O inter­molecular hydrogen bonds. The indole and creatinine units make a dihedral angle of 60.80 (4)°.

## Related literature

For the biological activity of isatin and its derivatives, see: Pandeya *et al.* (2005 ▶); The endogenous oxindoles 5-hydroxy­oxindole and isatin are anti­proliferative and proapoptotic, see: Cane *et al.* (2000 ▶). For the *in vitro* cytotoxicity evaluation of some substituted isatin derivatives, see: Vine *et al.* (2007 ▶). For 2-indol-3-yl-methyl­enequinuclidin-3-ols and NADPH oxidase activity, see: Sekhar *et al.* (2003 ▶) and for novel substituted (*Z*)-2-(*N*-benzyl­indol-3-ylmethyl­ene)quinuclidin-3-one and (*Z*)-(±)-2-(*N*-benzyl­indol-3-yl methyl­ene)quinuclidin-3-ol derivatives as potent thermal sensitizing agents, see: Sonar *et al.* (2007 ▶). For the crystal and mol­ecular structure of isatin, see: Frolova *et al.* (1988 ▶), for 3-(2-amino-1-methyl-4-oxo-4,5-dihydro-1*H*-imidazol-5-yl)-3-hydroxy­indolin-2-one monohydrate, see: Penthala *et al.* (2009 ▶) and for 1,1′-diacetyl-3-hydr­oxy-2,2′,3,3′-tetra­hydro-3,3′-bi(1*H*-indole)-2,2′-dione, see: Usman *et al.* (2002 ▶). For the aldol condensation enolate mechanism *via* a six-membered transition state, see: Zimmerman & Traxler (1957 ▶). 
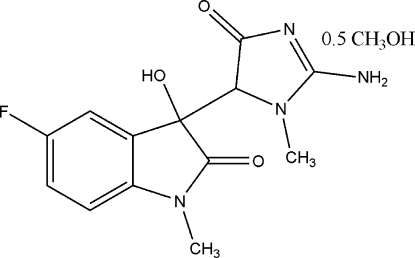


## Experimental

### 

#### Crystal data

C_13_H_13_FN_4_O_3_·0.5CH_4_O
*M* *_r_* = 308.30
Monoclinic, 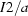

*a* = 14.3088 (3) Å
*b* = 10.7900 (2) Å
*c* = 18.1286 (5) Å
β = 107.676 (1)°
*V* = 2666.77 (10) Å^3^
*Z* = 8
Cu *K*α radiation
μ = 1.04 mm^−1^
*T* = 90 K
0.15 × 0.03 × 0.02 mm

#### Data collection

Bruker X8 Proteum diffractometer
Absorption correction: multi-scan (*SADABS* in *APEX2*; Bruker, 2006 ▶) *T*_min_ = 0.805, *T*_max_ = 0.979
19606 measured reflections
2448 independent reflections
2196 reflections with *I* > 2σ(*I*)
*R*_int_ = 0.041

#### Refinement

*R*[*F* ^2^ > 2σ(*F* ^2^)] = 0.035
*wR*(*F* ^2^) = 0.092
*S* = 1.04
2448 reflections
211 parameters
H-atom parameters constrained
Δρ_max_ = 0.25 e Å^−3^
Δρ_min_ = −0.27 e Å^−3^

### 

Data collection: *APEX2* (Bruker, 2006 ▶); cell refinement: *SAINT* (Bruker, 2006 ▶); data reduction: *SAINT*; program(s) used to solve structure: *SHELXS97* (Sheldrick, 2008 ▶); program(s) used to refine structure: *SHELXL97* (Sheldrick, 2008 ▶); molecular graphics: *XP* in *SHELXTL* (Sheldrick, 2008 ▶); software used to prepare material for publication: *SHELXL97* and local procedures.

## Supplementary Material

Crystal structure: contains datablocks global, I. DOI: 10.1107/S1600536809043797/hg2556sup1.cif

Structure factors: contains datablocks I. DOI: 10.1107/S1600536809043797/hg2556Isup2.hkl

Additional supplementary materials:  crystallographic information; 3D view; checkCIF report

## Figures and Tables

**Table 1:** Hydrogen-bond geometry (Å, °)

*D*—H⋯*A*	*D*—H	H⋯*A*	*D*⋯*A*	*D*—H⋯*A*
O2—H2⋯N2^i^	0.84	1.97	2.8074 (16)	171
N3—H3*A*⋯O3^ii^	0.88	2.25	3.1265 (16)	177
N3—H3*B*⋯O1^iii^	0.88	2.12	2.8490 (17)	140
N3—H3*B*⋯F1^iv^	0.88	2.45	2.8743 (14)	110
O1*S*—H1*S*4⋯O3	0.84	2.01	2.846 (3)	171

Symmetry codes: (i) 
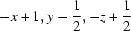
; (ii) 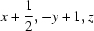; (iii) 
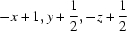
; (iv) 
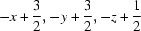
.

**Fig. 1.**
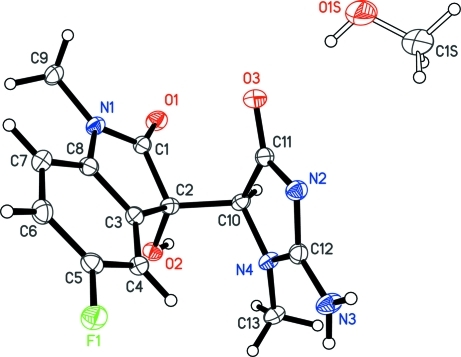
A view of the molecule with the atom numbering scheme. Displacement ellipsoids are drawn at the 50% probability level.

## supplementary crystallographic information

### Comment

Isatin analogs display diverse biological activities, (Pandeya *et al.*, 2005; Cane *et al.*, 2000 and Vine *et al.*, 2007). In continuation of our work on radiosensitizers (Sekhar *et al.*, 2003; Sonar *et al.*, 2007), we focused on the design, synthesis and structural analysis of a series of 3-(2-amino-1-methyl-4-oxo-4,5-dihydro-1*H*-imidazol-5-yl)-3 -hydroxyindolin-2-one analogs with different substituents on the indole moiety. The main aim of X-ray analysis of the title compound was to confirm the stereochemistry of the molecule and to obtain detailed information on the structural conformation, which may be useful in structure-activity relationship (SAR) analysis. The title compound was prepared by the aldol condensation of 5-fluoro-*N*-methyl indol-2,3-dione with 2-amino- 1-methyl-1*H*-imidazol-4(5*H*)-one (creatinine) in the presence of sodium acetate in acetic acid under microwave irradiation. The compound was crystallized from methyl alcohol. This aldol condensation reaction proceeds by the formation of the E-enolate, as per the Zimmerman-Traxler model (Zimmerman & Traxler, 1957). The molecular structure and the atom-numbering scheme are shown in Fig.1. The isatin ring is planar with r.m.s. deviation of 0.0232 (11) Å and the creatinine ring has r.m.s. deviation of 0.0307 (8) Å. with bond distances and angles comparable with those previously reported for other isatin derivatives (Frolova *et al.*, 1988; Usman *et al.*, 2002 and Penthala *et al.* (2009). The indole and creatinine moieties make a dihedral angle of 60.80 (4) °. Intermolecular O—H···N, N—H···O, N—H···F and O—H···O hydrogen bonds stabilize the crystal structure and form a supramolecular aggregation.

### Experimental

A mixture of 5-fluoro-*N*-methyl isatin (1 mmol), creatinine (1.1 mmol) and sodium acetate (1.2 mmol) in acetic acid (1 ml) was irradiated in a domestic microwave oven for 30 sec with intermittent cooling every 5 sec. The reaction mixture was allowed to cool to room temperature, 10 ml of saturated sodium bicarbonate solution was added, and the mixture was stirred for 10 minutes. The precipitate thus obtained was collected by filtration, washed with cold water and dried, to afford the crude product. Crystallization from methyl alcohol gave a white crystalline product of 3-(2-amino-1-methyl-4-oxo-4,5-dihydro-1*H*-imidazol-5-yl)- 5-fluoro-3-hydroxy-1-methylindolin-2-one methanolate, which was suitable for X-ray analysis. ^1^H NMR (DMSO-d_6_): δ 3.05 (*s*, 3H), 3.18 (*s*, 3H), 4.11 (*s*, 1H), 6.58 (*s*, 1H, OH), 6.85–6.89 (*dd*, J=2.7 Hz, J=5.4 Hz, 1H), 6.93–6.97 (*dd*, J=2.7 Hz, J=4.2 Hz, 1H), 7.11–7.18 (*m*, 1H), 7.42 (*bs*, 1H, NH), 7.72 (*bs*, 1H, NH), p.p.m.; ^13^C NMR (DMSO-d_6_): δ 26.08, 32.76, 48.62, 69.87, 76.29, 109.14, 109.25, 111.16, 111.50, 115.42, 115.72, 128.84, 128.95, 140.23, 156.35, 159.49, 171.98, 173.97, 181.85 p.p.m..

### Refinement

H atoms were found in difference Fourier maps and subsequently placed in idealized positions with constrained distances of 0.98Å (RCH_3_), 1.00 Å (R_3_CH), 0.95 Å (C_Ar_H), 0.84 Å (O—H), 0.88 Å (N—H), and with U_iso_(H) values set to either 1.2U_eq_ or 1.5U_eq_ (RCH_3_, OH) of the attached atom.

The presence of difference map peaks in the vicinity of the 2-fold axis at *ca* (1/4,0.62,0) were consistent with a disordered methanol solvent molecule. This methanol was modelled at half occupancy, such that application of the 2-fold site symmetry generates a full occupancy for the site. There is an O—H···O hydrogen-bonding interaction between this methanol and O3 of the main molecule (see Table 1).

### Crystal data

**Table:**

C_13_H_13_FN_4_O_3_·0.5CH_4_O	*F*(000) = 1288
*M**_r_* = 308.30	*D*_x_ = 1.536 Mg m^−^^3^
Monoclinic, *I*2/*a*	Cu *K*α radiation, λ = 1.54178 Å
Hall symbol: -I 2ya	Cell parameters from 9961 reflections
*a* = 14.3088 (3) Å	θ = 4.8–68.5°
*b* = 10.7900 (2) Å	µ = 1.04 mm^−^^1^
*c* = 18.1286 (5) Å	*T* = 90 K
β = 107.676 (1)°	Rod, colourless
*V* = 2666.77 (10) Å^3^	0.15 × 0.03 × 0.02 mm
*Z* = 8	

### Data collection

**Table:**

Bruker X8 Proteum diffractometer	2448 independent reflections
Radiation source: fine-focus rotating anode	2196 reflections with *I* > 2σ(*I*)
graded multilayer optics	*R*_int_ = 0.041
Detector resolution: 5.6 pixels mm^-1^	θ_max_ = 68.5°, θ_min_ = 4.8°
φ and ω scans	*h* = −17→17
Absorption correction: multi-scan (*SADABS* in *APEX2*; Bruker, 2006)	*k* = −13→13
*T*_min_ = 0.805, *T*_max_ = 0.979	*l* = −17→21
19606 measured reflections	

### Refinement

**Table:**

Refinement on *F*^2^	Primary atom site location: structure-invariant direct methods
Least-squares matrix: full	Secondary atom site location: difference Fourier map
*R*[*F*^2^ > 2σ(*F*^2^)] = 0.035	Hydrogen site location: inferred from neighbouring sites
*wR*(*F*^2^) = 0.092	H-atom parameters constrained
*S* = 1.04	*w* = 1/[σ^2^(*F*_o_^2^) + (0.0452*P*)^2^ + 3.1985*P*] where *P* = (*F*_o_^2^ + 2*F*_c_^2^)/3
2448 reflections	(Δ/σ)_max_ < 0.001
211 parameters	Δρ_max_ = 0.25 e Å^−^^3^
0 restraints	Δρ_min_ = −0.27 e Å^−^^3^

### Special details

**Table:**

Geometry. All e.s.d.'s (except the e.s.d. in the dihedral angle between two l.s. planes) are estimated using the full covariance matrix. The cell e.s.d.'s are taken into account individually in the estimation of e.s.d.'s in distances, angles and torsion angles; correlations between e.s.d.'s in cell parameters are only used when they are defined by crystal symmetry. An approximate (isotropic) treatment of cell e.s.d.'s is used for estimating e.s.d.'s involving l.s. planes.
Refinement. Refinement of *F*^2^ against ALL reflections. The weighted *R*-factor *wR* and goodness of fit *S* are based on *F*^2^, conventional *R*-factors *R* are based on *F*, with *F* set to zero for negative *F*^2^. The threshold expression of *F*^2^ > 2σ(*F*^2^) is used only for calculating *R*-factors(gt) *etc*. and is not relevant to the choice of reflections for refinement. *R*-factors based on *F*^2^ are statistically about twice as large as those based on *F*, and *R*- factors based on ALL data will be even larger.

### Fractional atomic coordinates and isotropic or equivalent isotropic displacement parameters (Å^2^)

**Table:**

	*x*	*y*	*z*	*U*_iso_*/*U*_eq_	Occ. (<1)
F1	0.71683 (6)	0.82226 (8)	0.40909 (5)	0.0226 (2)	
O1	0.34944 (7)	0.34900 (10)	0.34227 (6)	0.0220 (2)	
N1	0.40050 (8)	0.53388 (11)	0.40330 (7)	0.0180 (3)	
C1	0.40738 (10)	0.43524 (13)	0.35922 (8)	0.0171 (3)	
O2	0.56972 (7)	0.35213 (9)	0.37870 (6)	0.0182 (2)	
H2	0.5465	0.2822	0.3628	0.027*	
N2	0.48670 (8)	0.61111 (11)	0.17484 (7)	0.0186 (3)	
C2	0.50466 (10)	0.44565 (13)	0.33829 (8)	0.0164 (3)	
O3	0.34268 (7)	0.56370 (9)	0.20215 (6)	0.0207 (2)	
N3	0.64656 (9)	0.58544 (12)	0.16571 (8)	0.0230 (3)	
H3A	0.7006	0.5411	0.1762	0.028*	
H3B	0.6417	0.6549	0.1393	0.028*	
C3	0.54173 (10)	0.57182 (13)	0.37146 (8)	0.0162 (3)	
N4	0.57662 (8)	0.44285 (11)	0.22994 (7)	0.0168 (3)	
C4	0.62309 (10)	0.64045 (13)	0.37024 (8)	0.0168 (3)	
H4	0.6668	0.6122	0.3436	0.020*	
C5	0.63765 (10)	0.75245 (14)	0.40985 (8)	0.0181 (3)	
C6	0.57663 (11)	0.79772 (14)	0.44928 (8)	0.0205 (3)	
H6	0.5904	0.8747	0.4758	0.025*	
C7	0.49419 (11)	0.72920 (14)	0.44984 (8)	0.0206 (3)	
H7	0.4503	0.7582	0.4762	0.025*	
C8	0.47874 (10)	0.61742 (13)	0.41062 (8)	0.0174 (3)	
C9	0.32581 (11)	0.54673 (15)	0.44237 (9)	0.0227 (3)	
H9A	0.2715	0.5971	0.4105	0.034*	
H9B	0.3544	0.5872	0.4926	0.034*	
H9C	0.3013	0.4646	0.4503	0.034*	
C10	0.48606 (10)	0.43188 (13)	0.25013 (8)	0.0158 (3)	
H10	0.4521	0.3519	0.2308	0.019*	
C11	0.42843 (10)	0.54179 (13)	0.20572 (8)	0.0166 (3)	
C12	0.57275 (10)	0.54783 (13)	0.18962 (8)	0.0174 (3)	
C13	0.65283 (10)	0.34938 (13)	0.24071 (9)	0.0200 (3)	
H13A	0.6606	0.3276	0.1904	0.030*	
H13B	0.6344	0.2753	0.2644	0.030*	
H13C	0.7149	0.3822	0.2746	0.030*	
O1S	0.2149 (2)	0.6430 (4)	0.05671 (19)	0.0769 (13)	0.50
H1S4	0.2574	0.6239	0.0984	0.115*	0.50
C1S	0.2489 (9)	0.6226 (3)	−0.0003 (6)	0.0403 (9)	0.5
H1S1	0.3121	0.6649	0.0091	0.060*	0.50
H2S1	0.2026	0.6540	−0.0482	0.060*	0.50
H3S1	0.2580	0.5332	−0.0051	0.060*	0.50

### Atomic displacement parameters (Å^2^)

**Table:**

	*U*^11^	*U*^22^	*U*^33^	*U*^12^	*U*^13^	*U*^23^
F1	0.0206 (4)	0.0228 (5)	0.0262 (5)	−0.0075 (3)	0.0100 (3)	−0.0027 (3)
O1	0.0189 (5)	0.0224 (5)	0.0266 (6)	−0.0049 (4)	0.0098 (4)	−0.0021 (4)
N1	0.0153 (6)	0.0215 (6)	0.0188 (6)	−0.0016 (5)	0.0076 (5)	−0.0022 (5)
C1	0.0156 (7)	0.0204 (7)	0.0158 (7)	0.0003 (5)	0.0056 (5)	0.0016 (5)
O2	0.0172 (5)	0.0165 (5)	0.0200 (5)	0.0007 (4)	0.0043 (4)	0.0002 (4)
N2	0.0174 (6)	0.0192 (6)	0.0203 (6)	0.0003 (5)	0.0073 (5)	0.0020 (5)
C2	0.0138 (6)	0.0176 (7)	0.0180 (7)	−0.0001 (5)	0.0052 (5)	0.0006 (5)
O3	0.0153 (5)	0.0223 (5)	0.0246 (5)	0.0007 (4)	0.0065 (4)	0.0000 (4)
N3	0.0202 (6)	0.0225 (6)	0.0297 (7)	0.0015 (5)	0.0129 (5)	0.0070 (5)
C3	0.0160 (7)	0.0175 (7)	0.0145 (6)	0.0016 (5)	0.0039 (5)	0.0012 (5)
N4	0.0158 (6)	0.0166 (6)	0.0206 (6)	0.0013 (4)	0.0096 (5)	0.0014 (5)
C4	0.0152 (6)	0.0196 (7)	0.0158 (7)	0.0008 (5)	0.0050 (5)	0.0004 (5)
C5	0.0158 (6)	0.0197 (7)	0.0180 (7)	−0.0029 (5)	0.0037 (5)	0.0029 (5)
C6	0.0231 (7)	0.0192 (7)	0.0184 (7)	−0.0004 (6)	0.0051 (6)	−0.0032 (6)
C7	0.0199 (7)	0.0241 (7)	0.0190 (7)	0.0013 (6)	0.0078 (6)	−0.0028 (6)
C8	0.0147 (6)	0.0211 (7)	0.0160 (7)	0.0000 (5)	0.0039 (5)	0.0012 (5)
C9	0.0184 (7)	0.0321 (8)	0.0206 (7)	−0.0025 (6)	0.0103 (6)	−0.0039 (6)
C10	0.0133 (7)	0.0168 (7)	0.0185 (7)	−0.0015 (5)	0.0069 (5)	−0.0010 (5)
C11	0.0160 (7)	0.0176 (7)	0.0160 (7)	−0.0003 (5)	0.0044 (5)	−0.0027 (5)
C12	0.0181 (7)	0.0184 (7)	0.0160 (7)	−0.0015 (5)	0.0058 (5)	−0.0016 (5)
C13	0.0183 (7)	0.0197 (7)	0.0245 (7)	0.0034 (6)	0.0102 (6)	0.0008 (6)
O1S	0.0548 (19)	0.136 (4)	0.0458 (18)	0.060 (2)	0.0247 (16)	0.038 (2)
C1S	0.0351 (15)	0.0419 (18)	0.0384 (17)	0.012 (4)	0.0029 (13)	−0.033 (4)

### Geometric parameters (Å, °)

**Table:**

F1—C5	1.3641 (16)	C4—C5	1.389 (2)
O1—C1	1.2220 (17)	C4—H4	0.9500
N1—C1	1.3525 (19)	C5—C6	1.375 (2)
N1—C8	1.4117 (18)	C6—C7	1.395 (2)
N1—C9	1.4570 (18)	C6—H6	0.9500
C1—C2	1.5539 (19)	C7—C8	1.383 (2)
O2—C2	1.4168 (16)	C7—H7	0.9500
O2—H2	0.8400	C9—H9A	0.9800
N2—C11	1.3606 (18)	C9—H9B	0.9800
N2—C12	1.3615 (18)	C9—H9C	0.9800
C2—C3	1.5175 (19)	C10—C11	1.5270 (19)
C2—C10	1.546 (2)	C10—H10	1.0000
O3—C11	1.2318 (17)	C13—H13A	0.9800
N3—C12	1.3214 (19)	C13—H13B	0.9800
N3—H3A	0.8800	C13—H13C	0.9800
N3—H3B	0.8800	O1S—C1S	1.288 (10)
C3—C4	1.386 (2)	O1S—H1S4	0.8400
C3—C8	1.395 (2)	C1S—H1S1	0.9800
N4—C12	1.3401 (19)	C1S—H2S1	0.9800
N4—C13	1.4543 (18)	C1S—H3S1	0.9800
N4—C10	1.4544 (17)		
			
C1—N1—C8	111.10 (12)	C7—C8—C3	122.81 (13)
C1—N1—C9	123.91 (12)	C7—C8—N1	127.11 (13)
C8—N1—C9	124.85 (12)	C3—C8—N1	110.08 (12)
O1—C1—N1	125.61 (13)	N1—C9—H9A	109.5
O1—C1—C2	125.69 (13)	N1—C9—H9B	109.5
N1—C1—C2	108.59 (11)	H9A—C9—H9B	109.5
C2—O2—H2	109.5	N1—C9—H9C	109.5
C11—N2—C12	105.90 (12)	H9A—C9—H9C	109.5
O2—C2—C3	109.77 (11)	H9B—C9—H9C	109.5
O2—C2—C10	110.25 (11)	N4—C10—C11	100.60 (11)
C3—C2—C10	115.20 (11)	N4—C10—C2	111.45 (11)
O2—C2—C1	108.60 (11)	C11—C10—C2	111.49 (11)
C3—C2—C1	101.49 (11)	N4—C10—H10	111.0
C10—C2—C1	111.09 (11)	C11—C10—H10	111.0
C12—N3—H3A	120.0	C2—C10—H10	111.0
C12—N3—H3B	120.0	O3—C11—N2	126.72 (13)
H3A—N3—H3B	120.0	O3—C11—C10	123.14 (13)
C4—C3—C8	119.69 (13)	N2—C11—C10	110.09 (11)
C4—C3—C2	131.87 (13)	N3—C12—N4	122.26 (13)
C8—C3—C2	108.42 (12)	N3—C12—N2	123.14 (13)
C12—N4—C13	124.37 (12)	N4—C12—N2	114.60 (12)
C12—N4—C10	108.28 (11)	N4—C13—H13A	109.5
C13—N4—C10	126.78 (11)	N4—C13—H13B	109.5
C3—C4—C5	116.85 (13)	H13A—C13—H13B	109.5
C3—C4—H4	121.6	N4—C13—H13C	109.5
C5—C4—H4	121.6	H13A—C13—H13C	109.5
F1—C5—C6	118.00 (13)	H13B—C13—H13C	109.5
F1—C5—C4	118.03 (12)	C1S—O1S—H1S4	109.5
C6—C5—C4	123.97 (13)	O1S—C1S—H1S1	109.5
C5—C6—C7	119.17 (13)	O1S—C1S—H2S1	109.5
C5—C6—H6	120.4	H1S1—C1S—H2S1	109.5
C7—C6—H6	120.4	O1S—C1S—H3S1	109.5
C8—C7—C6	117.51 (13)	H1S1—C1S—H3S1	109.5
C8—C7—H7	121.2	H2S1—C1S—H3S1	109.5
C6—C7—H7	121.2		
			
C8—N1—C1—O1	−179.11 (13)	C2—C3—C8—N1	−2.46 (15)
C9—N1—C1—O1	5.0 (2)	C1—N1—C8—C7	178.43 (14)
C8—N1—C1—C2	4.55 (15)	C9—N1—C8—C7	−5.7 (2)
C9—N1—C1—C2	−171.35 (12)	C1—N1—C8—C3	−1.40 (16)
O1—C1—C2—O2	−66.31 (17)	C9—N1—C8—C3	174.45 (13)
N1—C1—C2—O2	110.02 (12)	C12—N4—C10—C11	6.11 (14)
O1—C1—C2—C3	178.05 (13)	C13—N4—C10—C11	−165.51 (13)
N1—C1—C2—C3	−5.62 (14)	C12—N4—C10—C2	−112.18 (13)
O1—C1—C2—C10	55.09 (18)	C13—N4—C10—C2	76.20 (17)
N1—C1—C2—C10	−128.58 (12)	O2—C2—C10—N4	−60.19 (14)
O2—C2—C3—C4	68.20 (19)	C3—C2—C10—N4	64.70 (15)
C10—C2—C3—C4	−56.9 (2)	C1—C2—C10—N4	179.38 (11)
C1—C2—C3—C4	−177.03 (14)	O2—C2—C10—C11	−171.72 (10)
O2—C2—C3—C8	−110.02 (13)	C3—C2—C10—C11	−46.83 (16)
C10—C2—C3—C8	124.84 (13)	C1—C2—C10—C11	67.85 (14)
C1—C2—C3—C8	4.74 (14)	C12—N2—C11—O3	−176.78 (14)
C8—C3—C4—C5	0.7 (2)	C12—N2—C11—C10	5.73 (15)
C2—C3—C4—C5	−177.33 (13)	N4—C10—C11—O3	175.06 (13)
C3—C4—C5—F1	−179.53 (12)	C2—C10—C11—O3	−66.68 (17)
C3—C4—C5—C6	−0.1 (2)	N4—C10—C11—N2	−7.34 (14)
F1—C5—C6—C7	178.89 (12)	C2—C10—C11—N2	110.92 (13)
C4—C5—C6—C7	−0.6 (2)	C13—N4—C12—N3	−10.8 (2)
C5—C6—C7—C8	0.5 (2)	C10—N4—C12—N3	177.36 (13)
C6—C7—C8—C3	0.1 (2)	C13—N4—C12—N2	168.57 (12)
C6—C7—C8—N1	−179.68 (13)	C10—N4—C12—N2	−3.30 (16)
C4—C3—C8—C7	−0.8 (2)	C11—N2—C12—N3	177.68 (13)
C2—C3—C8—C7	177.70 (13)	C11—N2—C12—N4	−1.65 (16)
C4—C3—C8—N1	179.06 (12)		

### Hydrogen-bond geometry (Å, °)

**Table:**

*D*—H···*A*	*D*—H	H···*A*	*D*···*A*	*D*—H···*A*
O2—H2···N2^i^	0.84	1.97	2.8074 (16)	171
N3—H3A···O3^ii^	0.88	2.25	3.1265 (16)	177
N3—H3B···O1^iii^	0.88	2.12	2.8490 (17)	140
N3—H3B···F1^iv^	0.88	2.45	2.8743 (14)	110
O1S—H1S4···O3	0.84	2.01	2.846 (3)	171

Symmetry codes: (i) −*x*+1, *y*−1/2, −*z*+1/2; (ii) *x*+1/2, −*y*+1, *z*; (iii) −*x*+1, *y*+1/2, −*z*+1/2; (iv) −*x*+3/2, −*y*+3/2, −*z*+1/2.
